# A systematic review of locust phase polyphenism: from proximate mechanisms to ecology and management

**DOI:** 10.7717/peerj.21374

**Published:** 2026-07-07

**Authors:** Diriba Fufa Serdo, Zoltán Németh

**Affiliations:** 1 Department of Evolutionary Zoology and Human Biology, University of Debrecen, Debrecen, Hungary; 2 Department of Biology, Ambo University, Ambo, Ethiopia

**Keywords:** Climate change, Field-laboratory integration, Gut microbiome, Locust phase change, Molecular mechanisms, Phase polyphenism, Phenotypic plasticity, Systematic review

## Abstract

Locust phase polyphenism is a remarkable example of phenotypic plasticity, driven by population density to produce a dramatic shift between cryptic, solitarious and swarming, gregarious phenotypes. Despite over a century of research, the evidence base lacks systematic synthesis. We conducted a systematic review of 400 studies on locust phase polyphenism, integrating evidence across ecological, neurobiological, physiological, molecular, epigenetic, and microbial drivers. The results revealed that the evidence base is constrained by two critical limitations. First, severe taxonomic narrowness: 93.8% of studies focus on at least one of two model species (desert locust, *Schistocerca gregaria* and migratory locust, *Locusta migratoria*), with only 6.2% examining other locust species exclusively. Second, profound methodological disconnect: 84.5% of studies are laboratory-based, while field-only (6.0%) and integrated field-laboratory studies (6.2%) together constitute only 12.2% of the literature. Within this paradigm, mechanistic research has successfully mapped proximate pathways from tactile stimulation and serotonin/dopamine signaling to transcriptomic reprogramming and epigenetic regulation. However, direct species comparisons reveal fundamental divergence rather than conservation, challenging assumptions of universal mechanisms. Laboratory-derived pathways remain poorly integrated with field ecology—vegetation structure, nutritional geography, and climate dynamics—creating a translational impasse for predictive management. Emerging areas such as microbiome dynamics and transgenerational epigenetics require causal validation under ecologically relevant conditions. Reliance on the current narrow paradigm fundamentally limits both biological understanding and practical application. We propose a future research prioritizing: (1) phylogenetically broad comparative multi-omics to distinguish conserved cores from lineage-specific adaptations; (2) integrated field-laboratory experiments incorporating climate and landscape heterogeneity; (3) causal validation of emerging regulators in ecologically relevant contexts; and (4) translation of comparative insights into species-specific management tools through equitable partnerships with researchers and practitioners in outbreak-affected regions. Such integration is essential for developing predictive, sustainable management strategies in an era of global change.

## Introduction

Locusts (Orthoptera: Acrididae) exhibit a remarkable form of density-dependent phase polyphenism, transitioning dramatically between solitarious and gregarious phases in response to changes in population density (Uvarov, 1966; Pener & Simpson, 2009; Cullen et al., 2017). This polyphenism encompasses coordinated changes in behavior, morphology, physiology, neurobiology, chemical ecology, gene expression, and life history traits, ultimately scaling to population-level ecological dynamics (Simpson & Sword, 2008; Pener & Simpson, 2009; Guo & Kang, 2025). Solitarious locusts are cryptic, sedentary, and avoid conspecifics, whereas gregarious locusts are conspicuous, highly active, and strongly attracted to one another (Roessingh, Simpson & James, 1993; Gray et al., 2009; Pener & Simpson, 2009; Pocco et al., 2019). These gregarious locusts form massive migratory swarms capable of covering extensive geographical areas, decimating crops and often causing significant economic damage and food insecurity (Cullen et al., 2017; Zhang et al., 2019).

Consequently, understanding locust phase polyphenism is not merely a fundamental biological question but a critical prerequisite for developing proactive, predictive, and sustainable management strategies to safeguard global food security. Yet, effective prediction and intervention remain elusive, in part because the switch from solitary to gregarious behavior undermines traditional control tactics (Sword, Lecoq & Simpson, 2010). Beyond their impact as agricultural pests, locusts serve as a model system for studying phenotypic plasticity—the capacity of a single genotype to produce different phenotypes in response to the changing environment (West-Eberhard, 2003; Pfennig et al., 2010; Simpson, Sword & Lo, 2011). Their reversible phenotypic shifts provide critical insights into epigenetic regulation, neural modulation, hormonal control, and the evolution of complex traits (Ott & Rogers, 2010; Burrows, Rogers & Ott, 2011; Ernst et al., 2015). The urgency of locust phase polyphenism research has intensified with global change, as climate and land-use shifts may alter the geographic distribution, frequency, and dynamics of locust outbreaks (Zhang et al., 2019; Kimathi et al., 2020; Peng et al., 2020; Tang et al., 2023; Youngblood et al., 2023; Hosni et al., 2024; Mamo, Kinyanjui & Siewe, 2025). Over a century of research has generated a rich, multidisciplinary body of literature. Numerous narrative reviews have provided invaluable syntheses of locust phase polyphenism, integrating knowledge across its many facets (Kennedy, 1956; Pener, 1991; Pener & Yerushalmi, 1998; Simpson, McCaffery & HÄgele, 1999; Simpson, Sword & De Loof, 2005; Tanaka, 2006; De Loof et al., 2006; Simpson & Sword, 2009; Verlinden et al., 2009; Pener & Simpson, 2009; Sword, Lecoq & Simpson, 2010; Burrows, Rogers & Ott, 2011; Song, 2011; Tawfik, 2012; Wang & Kang, 2014; Ernst et al., 2015; Cullen et al., 2017; Ayali, 2019; Pflüger & Bräunig, 2021; Guo & Kang, 2025).

However, while previous reviews have provided invaluable narrative syntheses—and we gratefully acknowledge their foundational role in shaping our understanding—a systematic synthesis of the entire evidence base and its inherent biases and gaps has never been conducted. The present study addresses this fundamental gap by conducting the first systematic review of locust phase polyphenism. Our primary objectives are to: (1) quantify the taxonomic, methodological, and thematic composition of the literature; (2) identify critical evidence gaps and biases that constrain both biological understanding and translational application. Through this approach, we aim to provide a definitive, evidence-based roadmap to guide future interdisciplinary research and translate fundamental insight into more predictive, sustainable strategies for locust management.

## Methodology

### Literature search strategies

A systematic literature search was conducted following the PRISMA 2020 guidelines (Page et al., 2021). The search was conducted across three databases: PubMed, Web of Science Core Collection, and Scopus, covering all publications from 1921 through February 2025. The search strategy employed comprehensive nested Boolean operators, combining three core term categories following systematic approach to search strategy development (Bramer et al., 2018). These terms included: population terms (“locust” OR “Schistocerca” OR “*Locusta migratoria*” OR “Nomadacris” OR “Chortoicetes” OR “Austracris”), phenomenon terms; (“phase polyphenism” OR “phase polymorphism” OR “phase change” OR “phase transition” OR “phenotypic plasticity” OR “density-dependent” OR “gregari” OR “solitar”, “phase characteristic”), and; trait/outcome terms (“behavi” OR “morphometr” OR, “colo” OR “pheromone” OR “juvenile hormone” OR “corazonin” OR “serotonin” OR “transcriptom” OR “microbiome”). Search strings were adapted for each database’s specific syntax and controlled vocabularies. The exact search terms applied to each database are provided (File S1). Search results were restricted to studies published in English due to resource constraints. To identify additional records, backward and forward citation tracking was performed on key seminal articles identified during the initial search.

### Eligibility criteria

Studies were assessed against pre-defined eligibility criteria. Eligible studies included all locust species documented to exhibit density-dependent phase polyphenism. Eligible interventions or exposures encompassed any condition that alters or defines phase state, including but not limited to crowding, sensory stimulation, hormonal application, genetic or epigenetic manipulations, or dietary change. A required comparator was a control or contrasting group essential for defining phase differences, such as gregarious (crowded) *vs*. solitarious (isolated). Eligible outcomes included any measurable phase-related trait across behavioral, morphological, physiological, molecular, epigenetic, microbial, and life-history, and theoretical categories. Eligible study designs were primary research articles, including laboratory experiments, field experiments, and observational cohort studies. Theoretical models and simulations were included only if they tested hypotheses against primary empirical data. Narrative reviews, editorials, opinion pieces, and studies lacking primary data were excluded. Furthermore, primary studies on locusts that did not investigate phase-related traits or incorporate a density-dependent component as a central variable were excluded.

### Study selection, data extraction and risk of bias assessment

Following duplicate removal using Mendeley Reference Manager, two reviewers (D.F.S. and Z.N.) independently screened titles and abstracts, then full texts of potentially eligible records, with disagreements resolved through discussion. A standardized data extraction form was piloted in Excel and refined, after which data from all included studies were extracted by one reviewer and independently verified by the second. Extracted items included bibliometric data (authors, publication year, journal, country), study characteristics (locust species, life stage, primary research focus), methodological classification (laboratory-only, field-only, integrated field-laboratory, modeling, phylogenetic), and substantive findings for narrative synthesis. All extracted data were recorded in Excel for quantitative analysis. Risk of bias was appraised independently by both reviewers using the SYRCLE tool (Chen et al., 2014) for laboratory experiments and a customized framework for modeling studies—assessing transparency of model structure, input parameters, calibration, validation, and sensitivity analysis—with each study assigned a summary rating (low, moderate, or high) and disagreements resolved through discussion.

### Data synthesis methods

Due to anticipated heterogeneity in locust species, study designs, interventions, and outcome measures across the included studies, a quantitative meta-analysis was deemed inappropriate *a priori*. Therefore, we conducted a structured narrative synthesis following the Synthesis Without Meta-analysis reporting guideline. We conducted a qualitative assessment of risk of reporting bias due to missing results for each major synthesis. Given the methodological heterogeneity, statistical tests for asymmetry (*e.g*., funnel plots) were not feasible. Instead, the assessment was based on a qualitative evaluation of the literature landscape, where we examined the direction and consistency of effects, explored sources of heterogeneity (including risk of bias), and identified evidence gaps within each thematic area.

## Results

### Literature search and screening outcomes

**Figure 1:**
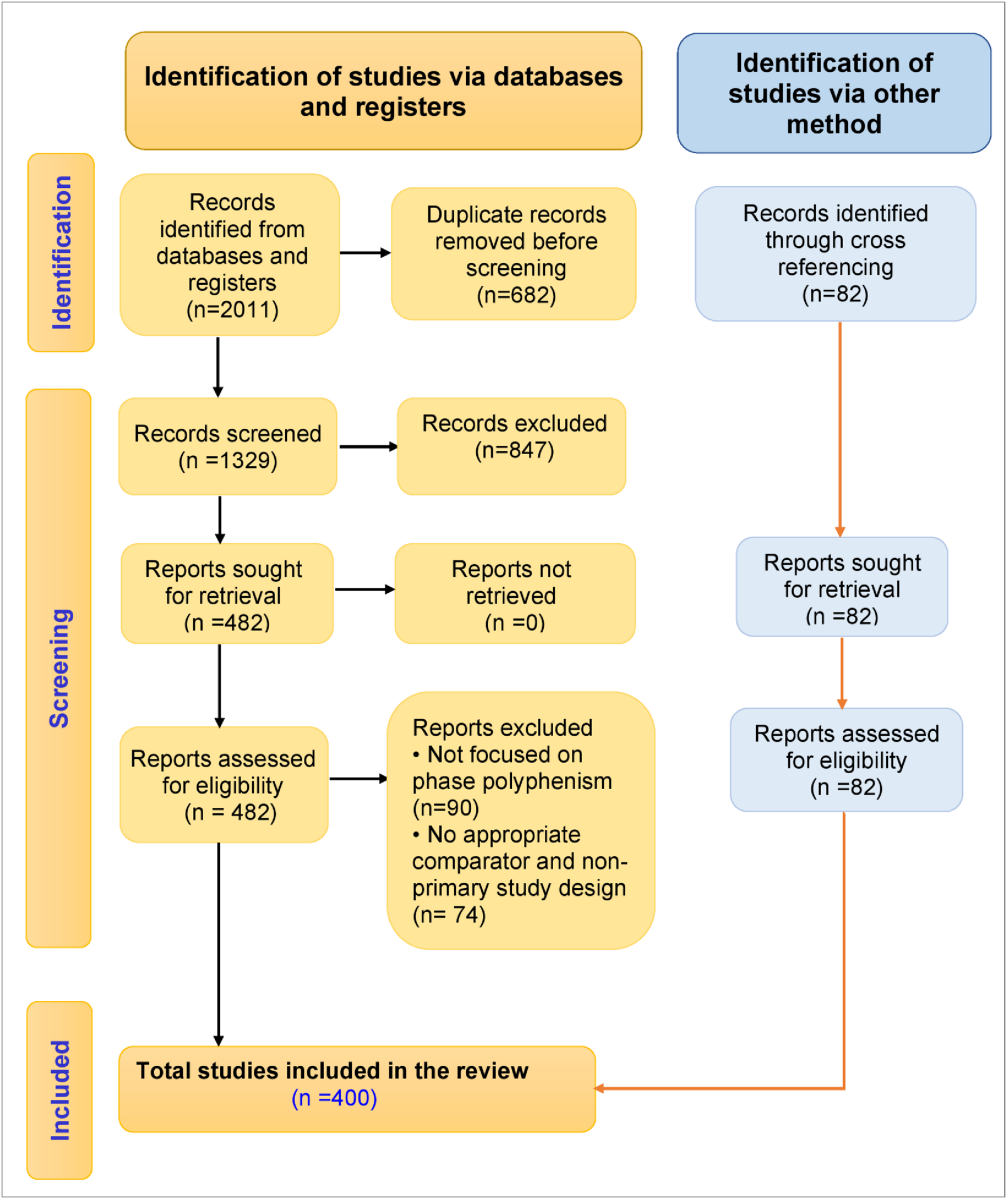
PRISMA flow diagram illustrating the systematic literature search and screening process. The four-stage process (identification, screening, eligibility, and inclusion) from the initial database search, and cross referencing to the final studies included in the bibliometric dataset.

The systematic literature search yielded 2,011 records. Following removal of duplicates (n = 682), 1,329 records proceeded to title and abstract screening, where 847 were excluded for not meeting preliminary eligibility criteria. The full texts of the remaining 482 records were obtained and evaluated, resulting in the exclusion of 164 records due to reasons such as lack of phase polyphenism focus, absence of a suitable comparator, or non-primary research design. Citation tracking of key seminal studies yielded an additional 82 studies, all of which passed the same two-stage screening process and were deemed eligible (Fig. 1). Consequently, the final synthesis comprised 400 unique primary studies investigating locust phase polyphenism, spanning from 1921 through February 2025 (see File S2 for the complete list).

### Characteristics of included studies

**Figure 2:**
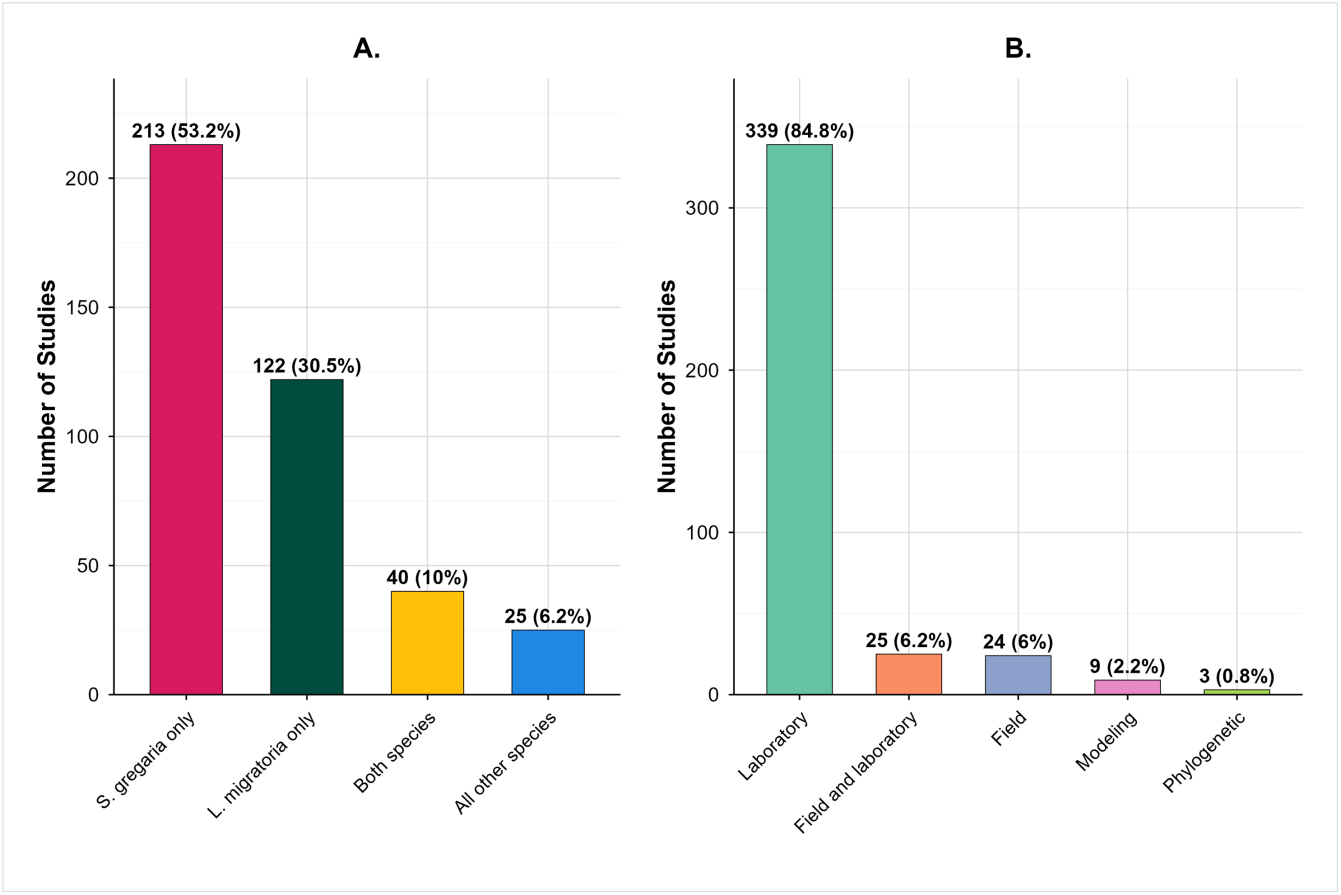
Taxonomic and methodological distribution of the 400 included studies. (A) Species focus showing strong bias toward *S. gregaria* (63.2%, *n* = 253) and *L. migratoria* (40.5%, *n* = 162), with only 6.2% (*n* = 25) examining other locust species. (B) Research approaches revealing predominance of laboratory-only studies (84.8%, *n* = 339), followed by field-only (6.0%, *n* = 24), combined field-lab (6.2%, *n* = 25), modeling (2.2%, *n* = 9), and phylogenetic approaches (0.8%, *n* = 3).

The 400 included studies exhibit substantial heterogeneity in species focus and methodology. Desert locust (*Schistocerca gregaria*) was the primary focus, appearing in 253 studies (63.2%), with 213 studies (53.2%) examining this species exclusively. Migratory locust (*Locusta migratoria)* was studied in 162 studies (40.5%), with 122 studies (30.5%) focusing solely on this species. Comparative studies examining both species together comprised 40 studies (10.0%). Combined, 375 studies (93.8%) involved at least one of these two model species. Only 25 studies (6.2%) focused exclusively on other locust species, including Australian plague locust (*Chortoicetes terminifera*) (Farrow, 1982; Gray et al., 2009; Cullen et al., 2010; Chapuis et al., 2011b, 2011a; Buhl et al., 2011; Cullen, Sword & Simpson, 2012; Robertson, Cease & Simpson, 2019), red locust (*Nomadacris septemfasciata)* (Michelmore & Allan, 1934; Pener, 1968; Dean, 1968; Franc et al., 2005; Lecoq, Chamouine & Luong-Skovmand, 2011; Bam, Conlong & Addison, 2024), South American locust *(Schistocerca cancellate)* (Pocco et al., 2019; Cease et al., 2023), the spur-throated locust *(Austracris guttulosa)* (Farrow, 1977; Elder, 1996), Central American locust *(Schistocerca piceifrons)* (Stahr & Seidelmann, 2016; Foquet & Song, 2021; Foquet, Castellanos & Song, 2021; Foquet et al., 2022), and Mongolian Locust (*Oedaleus asiaticus)* (Cease et al., 2010, 2017; Guo et al., 2022). Methodologically, laboratory-only studies accounted for 339 studies (84.8%). Field-only studies comprised 24 studies (6.0%). Studies combining both field and laboratory approaches represented 25 studies (6.2%), bringing the total with any field component to 49 studies (12.2%). Theoretical and modeling approaches accounted for nine studies (2.2%), with an additional three studies (0.8%) using phylogenetic methods (Fig. 2).

### Risk of bias within studies

The interpretation of findings on locust phase polyphenism must be considered in light of the methodological quality of the underlying studies. A risk of bias assessment was performed on all 400 included research articles, classifying each as “Low,” “Moderate,” or “High” risk based on study design, methodology, and reporting. The distribution of these classifications reveals distinct patterns related to study type and era of publication. The evidence base is dominated by studies with a “Low” risk of bias, which constitute the clear majority of the included literature. These are primarily modern laboratory experiments employing well-controlled, replicable designs, and include the landmark studies.

Studies assessed as having a “Moderate” risk of bias form a substantial minority. This category is characterized by two primary factors. First, it includes a large number of historically significant studies from the mid-20^th^ century. While pioneering for their time, these studies often lack the rigorous reporting standards expected today, such as detailed accounts of randomization, blinding, or statistical power analysis. Second, the “Moderate” category encompasses most field studies and some correlational laboratory work. The inherent challenges of controlling environmental confounders in field settings contribute to this classification, even when modern statistical methods are employed. A clear temporal trend is evident in the data: the proportion of “Low” risk studies has increased markedly over time. Research published from the 1990s onward predominantly meets modern standards for “Low” risk of bias, reflecting the widespread adoption of more rigorous experimental designs and analytical techniques. This temporal pattern indicates a field that has progressively strengthened its methodological foundations.

### Thematic synthesis

Analysis of the literature from the 1920s to 2025 reveals clear shifts in research themes, moving from descriptive natural history toward mechanistic molecular biology and, most recently, toward integrative, ecologically-grounded functional genomics. The foundational period (1920s to 1960s) established phase theory (Uvarov, 1921), behavioral foundations (Ellis, 1959a, 1959b, 1964; Ellis & Carlisle, 1961; Norris, 1962, 1963, 1964; Norris & Richards, 1970), morphometric phase indicators (Dirsh, 1951), transgenerational effects (Albrecht, 1957; Albrecht, Verdier & Blackith, 1959), and abiotic influences (Faure, 1932). The physiological and neuroethological regulation era (1970s to 2000s) focused on endocrine control and neuro-sensory processing, including reproductive diapause (Tanaka, Hakomori & Hasegawa, 1993), discovery of [His⁷]-corazonin (Tanaka & Pener, 1994; Tawfik et al., 1999), juvenile hormone regulation (Applebaum, Avisar & Heifetz, 1997), the neuroethological framework linking sensory stimuli to behavioral change (Simpson et al., 2001; Rogers et al., 2003), and characterization of aggregation pheromones (Fuzeau-Braesch et al., 1988; Torto et al., 1994, 1996; Njagi et al., 1996).

The molecular turn (2000s to 2015) ascended to genomics, transcriptomics, and epigenetics, including genomic resources (Kang et al., 2004), neuropeptide discovery (Clynen et al., 2002), receptor deorphanization (Verlinden et al., 2010), transcriptomic profiling (Chen et al., 2010), DNA methylation (Robinson et al., 2011; Falckenhayn et al., 2013), microRNA regulation (Wei et al., 2009), and serotonin/dopamine pathways (Rogers et al., 2004; Anstey et al., 2009; Ma et al., 2011), and the first phylogenetic analyses of phase polyphenism evolution (Song, 2005; Song & Wenzel, 2008). The integrative synthesis period (2015 to 2025) applies molecular approaches to higher-level questions through functional genomics, host-microbiota interactions, and integrative ecology, featuring gene editing validation (Li et al., 2016; Guo et al., 2020b), pheromone discovery (Guo et al., 2020b; Chang et al., 2023a), microbiome analysis (Lavy et al., 2019), nutritional geography (Cease et al., 2010, 2017), quantitative field ecology (Cisse et al., 2013; Cissé et al., 2016; Maeno, Piou & Ghaout, 2020), comparative transcriptomics demonstrating species-specificity (Yang et al., 2019a; Bakkali et al., 2024), and expanded evolutionary analyses integrating genomic and phenotypic data across multiple locust species (Song et al., 2017; Foquet, Castellanos & Song, 2021). Throughout these shifts, earlier research themes have persisted as essential context, but the frontier of discovery has moved decisively upward through successive levels of biological organization. The following sections synthesize the evidence according to this hierarchical framework, organizing studies by their primary level of analysis—from ecology through molecular regulation to emerging areas—while acknowledging that many studies bridge multiple levels.

#### Ecological triggers: density, resources, and environmental cues

Population density is the primary and most direct trigger for phase change (Uvarov & Hamilton, 1936; Ellis, 1959a; Simpson et al., 2001; Buhl et al., 2006). However, this density threshold is not fixed; rather, it is critically modulated by the spatial and nutritional structure of the environment. Clumped vegetation forces aggregation and lowers gregarization thresholds (Bouaïchi, Simpson & Roessingh, 1996; Collett et al., 1998; Despland & Simpson, 2000; Babah & Sword, 2004; Cisse et al., 2013, 2015a). Fractal analysis has demonstrated that vegetation clumping increases locust crowding and promotes gregarization across multiple spatial scales (Despland, 2003), while satellite-based studies have confirmed that landscape structure influences outbreak dynamics through its effects on resource abundance and fragmentation (Despland, Rosenberg & Simpson, 2004). Foraging models further support these findings, showing that food availability can lower the density requirements for group formation (Georgiou et al., 2021). Field studies have made particularly important contributions to understanding gregarization thresholds in natural populations. Research has established critical hopper densities of approximately 2.45 hoppers per square meter for observing gregarious individuals under field conditions (Cisse et al., 2013, 2015b), and has demonstrated that vegetation structure strongly influences these density thresholds (Cisse et al., 2013). A predictive model integrating adult density, vegetation status, and cover achieved 93.8% accuracy for phase prediction (Cissé et al., 2016), while recent work on *L. migratoria* has quantified behavioral phase differences through spatial distribution patterns in the field (Cissé et al., 2024).

Nutritional state further fine-tunes the density response. Protein-deficient diets increase movement and activity (Van der Zee, Behmer & Simpson, 2002; Bazazi et al., 2011; Cease et al., 2023), with low-protein availability increasing cannibalism-driven social interactions and thereby lowering the critical density for swarm formation (Bazazi et al., 2011). Nutritional geography research revealed that nitrogen-deficient soils promote protein-seeking behavior driving gregarization (Cease et al., 2010, 2017). However, the specific nutritional limitations vary among species: in *S. cancellata*, marching bands are carbohydrate-limited rather than protein-limited (Cease et al., 2023). Low-quality food can also increase extra molts and shift morphometrics toward gregarious traits (Maeno & Tanaka, 2011). Phase-specific differences in nutritional physiology are evident: gregarious nymphs overeat unbalanced diets and survive less well on poor foods (Simpson et al., 2002), whereas solitarious nymphs show greater food fidelity under nutritional challenge (Van der Zee, Behmer & Simpson, 2002).

Superimposed on these local ecological factors are broader climatic drivers that synchronize population dynamics over large geographic scales. Long-term dynamics are synchronized by climatic factors like rainfall, while abiotic cues such as humidity, temperature, substrate directly modulate phase-specific coloration (Faure, 1932; Dadd, 1961; Ellis, 1964; Tanaka, 2003; Maeno & Tanaka, 2007; Tanaka, Harano & Nishide, 2012; Nishide, Tanaka & Saeki, 2015; Ben Hamouda & Tanaka, 2016; Nishide, Suzuki & Tanaka, 2017; Sugahara & Tanaka, 2018). Temperature strongly affects melanin pigmentation and morphometric ratios (Dudley, 1964), and both egg temperature and moisture influence hatchling characteristics (Ben Hamouda & Tanaka, 2016). Mathematical models have identified temperature as a critical driver of population dynamics (Mamo, Kinyanjui & Siewe, 2025). Notably, species-specific hatching times are regulated by temperature, with phase effects present in *S. gregaria* but absent in *L. migratoria* (Nishide, Tanaka & Saeki, 2015; Nishide, Suzuki & Tanaka, 2017).

The adaptive significance of density-dependent responses extends beyond immediate behavioral and physiological adjustments to encompass shifts in survival strategies. Density-dependent color change functions as aposematic warning when locusts consume toxic plants (Sword et al., 2000), rather than serving as an intraspecific visual cue (Sword & Simpson, 2000). Solitarious nymphs avoid toxic plants while gregarious accept them, reflecting different antipredator strategies (Despland & Simpson, 2005a, 2005b). Theoretical models propose that clumped distributions disrupt predator efficiency (Reynolds et al., 2009), and provide density-dependent prophylaxis, enhancing pathogen resistance in crowded populations (Wilson et al., 2002; Elliot et al., 2003, 2005; Wang et al., 2013, 2022). Cannibalism risk has also been proposed as a selective force driving the evolution of behavioral polyphenism itself (Guttal et al., 2012).

These ecological pressures—resource distribution, nutritional stress, climatic variation, and predation risk—do not merely trigger immediate phase changes but also exert sustained selective forces that shape the genetic architecture of populations over evolutionary time. Population genetic studies have revealed that outbreaking populations exhibit stronger parental density-dependent phase change than non-outbreaking populations, demonstrating genetic variation for gregarization propensity (Chapuis et al., 2008). Parental crowding induces trans-generational effects on reproductive timing and offspring size (Chapuis et al., 2010), further indicating that phase-related traits have a genetic basis that can respond to selection. Recession (solitarious) populations retain genetic divergence and are not homogenized by past gregarious swarms, suggesting that solitarization of swarms may be limited (Ibrahim, Sourrouille & Hewitt, 2000; Ibrahim, 2001). Simulated population turnover during recession periods can maintain genetic structure despite mixing during plagues (Ibrahim, 2001), indicating that the ecological conditions prevailing during recessions may be as important as those during outbreaks in shaping population genetic structure.

#### Sensory initiation and neurobiological reprogramming

The rapid behavioral switch is initiated by tactile stimulation, though the key body regions vary by species: hind femur in *S. gregaria* (Hägele et al., 2000; Simpson et al., 2001; Rogers et al., 2003) *vs*. antennal contact in *C. terminifera* (Cullen et al., 2010). Visual and olfactory cues play modulatory roles (Ellis, 1959b, 1963a; Ellis & Pearce, 1962; Heifetz, Voet & Applebaum, 1996; Roessingh, Bouaïchi & Simpson, 1998; Despland, 2001; Lester et al., 2005; Tanaka, Harano & Nishide, 2012; Tanaka et al., 2016). Solitarious locusts possess approximately 30% more tactile hairs on the hind femur than gregarious locusts (Rogers et al., 2003). In *S. gregaria*, antennal tactile stimuli are necessary for maternal gregarization effects (Maeno, Tanaka & Harano, 2011).

Central nervous system processing shows distinct phase-specific patterns. Neurophysiological studies of the antennal lobe reveal that solitary nymphs have more responsive neurons with greater sensitivity to pheromone components (Anton & Hansson, 1996; Ignell, Anton & Hansson, 1998, 1999; Ochieng, Hallberg & Hansson, 1998; Ochieng’ & Hansson, 1999; Anton, Ignell & Hansson, 2002). Females have more specialist neurons, while males have more generalist neurons (Anton & Hansson, 1996). Solitary-reared adults possess more olfactory sensilla than crowd-reared adults (Ochieng, Hallberg & Hansson, 1998; Ochieng’ & Hansson, 1999). No anatomical differences exist in the antennal lobe, but physiological responses differ (Anton, Ignell & Hansson, 2002). Detailed studies of olfactory pathways have elucidated peripheral and central mechanisms mediating pheromone detection (Chang et al., 2023a, 2023b; Lehmann et al., 2024). Most locust odorant receptors are narrowly tuned, and odorant valence depends on phase, stage, and sex (Chang et al., 2023b). Sensory neuron membrane protein 1 (SNMP1) is critical for sensitive Phenylacetonitrile (PAN) detection (Lehmann et al., 2024). Gregarious locusts show synergistic olfactory processing of food and social odors (Petelski et al., 2024).

The neurochemical cascade executing this transformation reveals marked differences between model species. In *S. gregaria*, serotonin is necessary and sufficient for the rapid behavioral switch (Anstey et al., 2009; Rogers et al., 2014; Rogers & Ott, 2015), increasing nine-fold in thoracic ganglia within 4 h of crowding (Rogers et al., 2004). Different serotonergic neuron subsets mediate acute *vs*. long-term gregarization (Rogers & Ott, 2015). In *L. migratoria*, by contrast, serotonin modulates the transition rate but enhances solitariness rather than driving gregarization (Ma et al., 2011; Guo, Ma & Kang, 2013). A dopamine-octopamine-tyramine axis regulates the long-term behavioral state across species (Rogers et al., 2004; Alessi et al., 2014; Ma et al., 2015; Guo, Ma & Kang, 2015; Guo et al., 2018; Ma, Liu & Guo, 2019; Ma, Guo & Liu, 2020). Dopamine injection induces gregarization (Ma et al., 2011), with dopamine receptor 1 (Dop1) promoting gregariousness and dopamine receptor 2 (Dop2) promoting solitariness (Guo, Ma & Kang, 2015). Dop1 inhibits microRNA-9a (miR-9a) maturation, relieving repression of adenylyl cyclase 2 (AC2) to regulate olfactory attraction underlying aggregation (Guo et al., 2018). Isolation increases dopamine and serotonin while reducing octopamine, and dopamine injection induces solitarious-like behavior (Alessi et al., 2014). Dopamine sulfation promotes gregarious behavior and is conserved across species (Chen et al., 2022b). Octopamine receptor α (OARα) mediates attraction while tyramine-TAR mediates repulsion (Ma et al., 2015).

Octopamine receptor transcript levels are higher in gregarious locusts (Verlinden et al., 2010). Cellular retinaldehyde-binding protein (CRALBP), Translocator protein (TSPO), and retinoid X receptor-G protein α 14 (RXR-Gna14) signaling mediate olfactory attraction and repulsion (Ma, Liu & Guo, 2019; Ma & Liu, 2020; Ma, Guo & Liu, 2020). Odorant receptor deorphanization has advanced through international collaborative efforts (Li et al., 2016; Chang et al., 2023b; Lehmann et al., 2024). Orco knockout impairs olfactory responses but not activity or coloration (Li et al., 2016). Chemosensory protein (CSP) genes have undergone expansion *via* duplication in both model species, with most CSPs differentially expressed between phases. Notably, 14 orthologous CSP pairs are over-expressed in the gregarious phase of both species, suggesting conserved roles in phase change (Martín-Blázquez et al., 2017).

The kinetics of phase change are asymmetric and exhibit properties of long-term memory, but the specific time-course varies significantly among species. In the *L. migratoria*, behavioral transitions from solitarious to gregarious are slow, while the reverse is rapid (Ellis, 1963b; Harano et al., 2011, 2012; Guo et al., 2011; Ma et al., 2011; Guo, Ma & Kang, 2013, 2015). In contrast, the *S. gregaria* exhibits a quicker shift to gregarious behavior, but a slower transition back (Gillett, 1988; Roessingh, Simpson & James, 1993; Roessingh & Simpson, 1994; Bouaïchi, Roessingh & Simpson, 1995; Ott et al., 2012). *C. terminifera* shows intermediate dynamics, with behavioral phase change completing in either direction within days (Gray et al., 2009; Cullen et al., 2010). *S. piceifrons* exhibits kinetics similar to *L. migratoria* (rapid solitarization, slow gregarization) rather than its congener *S. gregaria*, suggesting phase-change dynamics are shaped by ecological pressures as much as by phylogeny (Foquet, Castellanos & Song, 2021; Foquet et al., 2022). Studies on the neural basis of gregarious behavior have revealed memory-like properties of phase change (Geva et al., 2010; Golov et al., 2018). A 30-min crowding event induces long-term behavioral change retained for 24 h and is sensitive to protein synthesis inhibition (Geva et al., 2010). Protein kinase A (PKA) activity is critical for initial gregarious behavior acquisition but not long-term expression (Ott et al., 2012).

This persistent shift is underpinned by extensive neural remodeling. Gregarious locusts have larger brains with expanded higher-processing centers (Ott & Rogers, 2010). The descending contralateral movement detector neuron habituates five times more strongly in solitarious locusts (Matheson, Rogers & Krapp, 2004; Rogers et al., 2007, 2010; Gaten et al., 2012) and has a more curved receptive field with reduced habituation in gregarious individuals (Rogers et al., 2010). Homeostatic plasticity maintains constant synaptic drive across phases (Rogers et al., 2007). The descending contralateral movement detector (DCMD) neuron shows circadian rhythmicity matching phase-specific activity times (Gaten et al., 2012). Solitarious locusts have enhanced high-frequency hearing for bat detection (Gordon et al., 2014). Gregarious locusts show stronger tritocerebral commissure giants (TCG) activity and flight initiation (Fuchs, Kutsch & Ayali, 2003; Ayali, Fuchs & Kutsch, 2004). Polarization-sensitive neurons for navigation show no phase differences (el Jundi & Homberg, 2012). Cognitive plasticity is integral, with phase change involving fundamental reprogramming of associative learning priorities. Aversive learning is phase-specific, and crowding blocks new aversive acquisition but not memory retrieval (Simões, Niven & Ott, 2013). Solitarious locusts show higher slow extensor tibiae (SETi) reflex gain and longer catalepsy, supporting cryptic lifestyle (Blackburn et al., 2010), and jump faster and farther but with higher energy cost (Rogers et al., 2016).

#### Chemical communication and coordination

Locusts employ phase-specific volatiles for collective behavior, but key pheromones are species-specific with sometimes opposing functions. In *L. migratoria*, 4-vinylanisole (4VA) acts as a key aggregation pheromone (Guo et al., 2020b; Yang et al., 2023b), specifically emitted by gregarious locusts, attracting both phases, detected by OR35, with release density-dependent (Guo et al., 2020a). 4VA accelerates gregarious behavior acquisition, and loss of perception prevents gregarization (Yang et al., 2023b). 4VA also promotes sexual maturation synchrony *via* the juvenile hormone and vitellogenin pathway (Chen et al., 2022a). Phenylacetonitrile reveals complete functional divergence between species. In *L. migratoria* nymphs, PAN serves as an anti-cannibalistic signal (Wei et al., 2019; Chang et al., 2023a) and olfactory aposematic signal converting to hydrogen cyanide (Wei et al., 2019). In *S. gregaria*, by contrast, PAN is a male-derived courtship inhibition pheromone (Seidelmann & Ferenz, 2002; Rono et al., 2008; Lehmann et al., 2024). PAN is twenty times higher in males, peaks at 2 to 3 weeks, and is absent in solitarious individuals (Amwayi et al., 2012). PAN production changes rapidly with density shifts (Deng et al., 1996) and has concentration-dependent dual functions: cohesive at low concentrations and repellent at high concentrations (Rono et al., 2008). Field experiments have demonstrated that PAN exposure can solitarize gregarious hopper bands (Bashir & Hassanali, 2010).

In *S. gregaria*, fecal volatiles like guaiacol and phenol, produced by gut bacteria *Pantoea agglomerans*, function as aggregation pheromones (Fuzeau-Braesch et al., 1988; Obeng-Ofori et al., 1994; Torto et al., 1994, 1996; Dillon, Vennard & Charnley, 2002). Three aromatic compounds including phenol, guaiacol, and veratrole promote aggregation (Fuzeau-Braesch et al., 1988). Two stage-specific pheromone systems exist: juvenile pheromone attracting nymphs, adult pheromone attracting adults (Obeng-Ofori, Torto & Hassanali, 1993). Fecal volatiles complement these systems (Obeng-Ofori et al., 1994). The adult aggregation pheromone is a male-produced blend (Torto et al., 1994). Solitarious adults lack PAN but respond to gregarious pheromone, aiding recruitment (Njagi et al., 1996).

Cuticular hydrocarbons serve as essential contact gregarizing signals across phases. Solitary *Locusta migratoria* produce higher proportions of longer-chain cuticular alkanes and heavier cuticular ethers compared to gregarious individuals (Genin et al., 1986; Genin, Jullien & Fuzeau-Braesch, 1987; Heifetz et al., 1997, 1998). Gregarious ethers are predominantly C_16_, while solitary ethers are C_18_ (Genin, Jullien & Fuzeau-Braesch, 1987). The hydrocarbon fraction induces gregarious behavior and elicits IP₃ responses in antennae (Heifetz et al., 1997). Gregarious hydrocarbon extract induces gregarious behavior; profiles differ quantitatively and change rapidly with density; species-specific (Heifetz et al., 1998). Cuticular contact cues are primary behavioral phase transition inducers (Heifetz, Voet & Applebaum, 1996).

Chemical signals also mediate maternal priming, with volatiles in oviposition sand and egg pod foam priming hatchlings for gregarious life (Mccaffery et al., 1998; Malual et al., 2001). Acetophenone and veratrole are major oviposition aggregation pheromone components (Rai et al., 1997). Three unsaturated ketones from sand mediate oviposition aggregation (Torto et al., 1999). Sand from gregarious females contains volatile C-8 ketones acting as primer pheromone (Malual et al., 2001). A small hydrophilic gregarizing factor exists in egg foam (Mccaffery et al., 1998). The primary gregarizing agent is an alkylated L-dopa analogue (Miller et al., 2008). Pheromone exposure induces proteomic changes in embryos (Khamis et al., 2015).

Pheromones regulate sexual maturation, with adult volatiles accelerating it (Loher, 1961; Norris, 1964; Mahamat et al., 1993; Mahamat, Hassanali & Odongo, 2000; Hiroyoshi et al., 2021) and nymphal volatiles delaying it. Early work established the role of pheromones in reproductive maturation (Norris, 1962, 1963, 1964; Norris & Pener, 1965; Norris & Richards, 1970). Mature male volatiles accelerate maturation in both sexes; the blend serves dual aggregation and maturation roles (Mahamat et al., 1993). PAN is critical for maturation acceleration (Mahamat, Hassanali & Odongo, 2000). Ovipositing females show strong gregarious cohesion mediated by contact pheromones (Norris, 1963; Norris & Richards, 1970). Gregarious males show enhanced sperm storage; adult volatiles promote, nymphal volatiles retard sperm storage (Hiroyoshi et al., 2021). Mature male extract accelerates maturation but does not induce yellowing (Amerasinghe, 1978a). Juvenile hormone I is more effective than juvenile hormone III for yellowing and sexual activity (Amerasinghe, 1978b). Solitarious females emit a volatile sex pheromone attracting males (Inayatullah, El Bashir & Hassanali, 1994). Both solitarious sexes use olfactory and visual cues for mate location; crowding enhances responsiveness (Ely et al., 2006). Solitarious males invest more in courtship; inter-phase mating success is asymmetric (Golov et al., 2018).

Dibutyl phthalate (DBP) is a phase-specific sex pheromone released by solitary females, crucial for low-density mate-finding (Cui et al., 2024). In *S. piceifrons*, male pheromone emission is density-dependent and mediates cryptic female choice (Stahr & Seidelmann, 2016). In *L. migratoria*, virgin gregarious females emit a phase-specific signal attracting only virgin gregarious males, creating an inter-phase mating barrier (Unni, Knaden & Hansson, 2024). Interspecific aggregation between *L. migratoria* and *S. gregaria* explains mixed hopper bands (Niassy et al., 1999). Recent behavioral work has elucidated collective motion mechanisms: locusts do not explicitly align with neighbors, vision is necessary and sufficient for coordinated marching, there is no density-dependent disorder-order transition as alignment depends on order rather than density, optomotor response is present but does not mediate social alignment, gregarious but not solitarious locusts align with virtual conspecifics, and behavior is explained by a vectorial representation or ring attractor cognitive model rather than classical self-propelled particle models (Sayin et al., 2025).

#### Physiological, morphological, and life-history reprogramming

The phase transition orchestrates a comprehensive physiological overhaul across multiple systems. While early studies emphasized hormonal control, recent research reveals a more complex integration of endocrine, direct signaling, and biochemical pathways. Coloration, one of the most visible phase traits, involves distinct regulatory pathways. Juvenile hormone (JH) regulates background homochromy, promoting the green/brown cryptic coloration of solitary locusts (Pener, 1967; Pener & Lazarovici, 1979; Injeyan & Tobe, 1981a; Tawfik et al., 1997b; Tanaka, 2000). In some species, [His⁷]-Corazonin (Crz) induces gregarious black patterning (Tawfik et al., 1999; Tanaka et al., 2002; Maeno, Gotoh & Tanaka, 2004; Sugahara et al., 2015, 2016, 2017), though its role is absent in some albino strains (Schoofs et al., 2000; Baggerman et al., 2001; Hoste et al., 2002; Rahman et al., 2003).

A recent discovery in *L. migratoria* revealed that the characteristic gregarious black patterning results not from melanin deposition but from a carotenoid-based mechanism. Crowding induces expression of β-carotene-binding protein (βCBP), which complexes with dietary β-carotene to form a red pigment. When superimposed on the solitary green background, this creates the black aposematic coloration through a “three primary colors” principle (Yang et al., 2019b). This density-responsive pathway involves phosphorylation of transcription factor ATF2, directly linking population density to precise spatial patterning (Kang et al., 2023). Complementary coloration mechanisms include: the presence of blue-green biliverdin pigment in solitary haemolymph, serving as a biochemical phase indicator (Goodwin & Srisukh, 1951; Mahamat, Hassanali & Munyinyi, 1997; Deng, 2002); and yellow protein (YP) expression in gregarious desert locusts (Sas et al., 2007), functioning as an intrasexual warning signal (Sugahara et al., 2018; Cullen et al., 2022).

The femur-to-head capsule width ratio (F/C) remains a reliable, species-general morphological phase index (Dirsh, 1951; Blackith, 1957; Tanaka, 2022, 2024). The role of juvenile hormone extends beyond coloration to complex behavioral modulation. While critical for reproduction and development, application of juvenile hormone analogs can suppress gregarious aggregation behavior, suggesting a role in promoting solitarization (Wiesel, Tappermann & Dorn, 1996; Applebaum, Avisar & Heifetz, 1997; Tawfik et al., 1997a; Guo et al., 2020a). This highlights the interconnected yet sometimes antagonistic relationships between endocrine pathways regulating different phase traits. A core life-history trade-off exists between reproductive strategies. Gregarious females produce fewer, larger, yolk-rich eggs, while solitarious females produce more, smaller eggs (Cheu, 1952; Albrecht, Verdier & Blackith, 1959; Injeyan & Tobe, 1981b; Maeno & Tanaka, 2008; Maeno et al., 2013; Chen et al., 2015a; Maeno, Piou & Ghaout, 2020; Zhao et al., 2021; Maeno, Piou & Leménager, 2023). This divergence reflects adaptive strategies: gregarious locusts invest in offspring quality for swarm migration, while solitary locusts maximize fecundity in stable environments.

Metabolic profiles shift dramatically between phases. Gregarious locusts are optimized for sustained migration, exhibiting enhanced adipokinetic hormone response and lipid metabolism (Ayali & Pener, 1992, 1995; Schneider & Dorn, 1994; Ayali, Pener & Girardie, 1996; Ogoyi, Osir & Olembo, 1996, 1998; Ayali, Golenser & Pener, 1996; Ayali et al., 1996; Du et al., 2022). Metabolomic analyses confirm distinct phase-specific profiles (Lenz et al., 2001; Wu et al., 2012), though this metabolic optimization carries trade-offs, including accelerated flight muscle ageing in gregarious individuals (Guo et al., 2023). Developmental plasticity is also phase-dependent, solitarious individuals are more prone to extra molting (Albrecht, 1957; Maeno & Tanaka, 2010a).

#### Molecular and epigenetic control mechanisms

##### Transcriptomic reprogramming

Crowding induces genome-wide transcriptional changes, with thousands of genes differentially expressed between phases (Kang et al., 2004; Wang et al., 2007, 2014; Chen et al., 2010; Badisco et al., 2011a, 2011b; Jiang et al., 2012; Lee et al., 2018; Bakkali & Martín-Blázquez, 2018). Early transcriptomic studies in *S. gregaria* and *L. migratoria* identified phase-specific expression patterns across multiple tissues, revealing coordinated regulation of genes involved in chemosensation, metabolism, cuticle formation, and neuropeptide signaling (Kang et al., 2004; Wang et al., 2007; Chen et al., 2010). A pivotal advance came from direct comparative transcriptomics between the two model species. Bakkali & Martín-Blázquez (2018) demonstrated that gene expression profiles cluster more strongly by species than by phase, with only a small set of genes, termed the PhaseCore, showing conserved phase-related expression across both species. This finding was subsequently confirmed and extended (Yang et al., 2019a; Bakkali et al., 2024), revealing that the majority of transcriptional responses to crowding are species-specific. This architecture, in which conserved gregarious phenotypes are achieved through divergent molecular means, explains why behavioral and morphological convergence across locust species masks profound mechanistic differences at the molecular level.

Among the conserved elements are chemosensory protein genes, which have undergone lineage-specific expansion *via* duplication in both model species. Most CSPs are differentially expressed between phases, and critically, 14 orthologous CSP pairs are over-expressed in the gregarious phase of both *S. gregaria* and *L. migratoria*, suggesting a conserved role in chemosensory adaptation to crowded conditions (Martín-Blázquez et al., 2017). Similarly, genes involved in dopamine synthesis and signaling show phase-related expression in both species, though the specific isoforms and regulatory networks differ (Guo et al., 2018; Zhang et al., 2020). Temporal transcriptomic studies have further revealed that phase change involves sequential waves of gene expression. Immediate early genes, including transcription factors and hormone receptors, are upregulated within hours of crowding, followed by delayed expression of effector genes such as cuticle proteins and metabolic enzymes (Wang et al., 2014; Guo et al., 2016). This temporal architecture mirrors the behavioral kinetics of gregarization: rapid initial behavioral shift followed by slower morphological and physiological consolidation.

##### Epigenetic regulation of phase change

Epigenetic mechanisms, including DNA methylation, histone modifications, and non-coding RNAs (lncRNA), are dynamically regulated by density and are critical for plasticity (Wei et al., 2009; Robinson et al., 2011, 2016; Falckenhayn et al., 2013; Mallon, Amarasinghe & Ott, 2016; He et al., 2016, 2022; Guo et al., 2016; Zhang et al., 2020; Hou et al., 2020; Li et al., 2020a; Yang et al., 2023a). Functional studies demonstrate that knockdown of DNA methyltransferase three reduces gregarious locomotion (Hou et al., 2020), and the lncRNA PAHAL promotes gregarization by regulating dopamine synthesis (Zhang et al., 2020; Li et al., 2020a). These findings establish that epigenetic regulation is not merely correlated with phase change but functionally required for its expression. The transcriptomic and epigenetic evidence together present a dual picture: extensive lineage-specific divergence in gene expression overlaid on a small core of conserved elements, with dynamic epigenetic regulation orchestrating the plastic response to crowding. This integrated molecular architecture explains why interventions targeting phase change may need to be species-specific, as the molecular targets of opportunity differ even when the phenotypic outcome is shared, while also identifying conserved elements such as specific CSPs or dopamine pathway components that might serve as broader targets if their functional roles prove essential across species.

#### Transgenerational effects and immunological adaptation

Phase state can be transmitted across generations *via* non-genetic mechanisms. Crowding experience of adults directly influences offspring phase (Ellis, 1959a; Islam et al., 1994a, 1994b; Bouaïchi & Simpson, 2003; Tanaka & Maeno, 2006; Chapuis et al., 2008, 2010; Maeno & Tanaka, 2010b; Chen et al., 2015b). This maternal effect involves the provisioning of larger eggs, a mechanism associated with gregarizing factors initially identified in egg foam (Miller et al., 2008; Mccaffery et al., 1998) and later linked to egg size itself (Maeno & Tanaka, 2008, 2009a, 2010b). Species-specificity is evident in the underlying mechanisms. Increasing juvenile hormone titers shifts progeny toward solitarious traits in *L. migratoria* (Ben Hamouda & Tanaka, 2016), but does not directly regulate phase-specific traits in *S. gregaria*, where direct crowding signals are paramount (Maeno & Tanaka, 2009b). Gregarious locusts also exhibit density-dependent prophylaxis—enhanced immune investment conferring greater resistance to fungal pathogens like *Metarhizium anisopliae* (Wilson et al., 2002; Wang et al., 2013, 2022). Conversely, infection by pathogens like the microsporidian *Paranosema locustae* and *Metarhizium anisopliae* can shift phase-related behavior and morphometrics towards solitarious traits (Elliot et al., 2003; Feng et al., 2015; Li et al., 2020b).

#### Emerging role of the gut microbiome

A clear phase-specific dichotomy exists in gut microbiota. Gregarious locusts are characterized by a transient dominance of *Weissella* (Firmicutes), which is horizontally transmitted upon crowding, while solitary locusts maintain a more stable core microbiome dominated by Proteobacteria (Lavy et al., 2019, 2022). However, field-collected gregarious locusts can share a stable core microbiome with solitary ones, suggesting the laboratory dynamic may be an artifact of density-facilitated transmission in simplified environments (Lavy et al., 2019). The microbiome both influences and is influenced by phase. Gut bacteria produce aggregation pheromone components (Dillon, Vennard & Charnley, 2002), and phase affects reproductive tract microbiota (Lavy et al., 2020). This creates a feedback loop where females vertically transmit symbionts, while environmental resource distribution facilitates the social interactions necessary for microbial transmission.

## Discussion

### General interpretation of the results

This systematic review synthesizes over a century of research on locust phase polyphenism, revealing a field that has generated profound mechanistic insight within a narrow empirical paradigm. The evidence base shows two dominant patterns: an overwhelming focus on two model species—*S. gregaria* and *L. migratoria*, which together appear in 93.8% of studies—and a methodological reliance on controlled laboratory experiments, accounting for 84.8% of all included studies. While this approach has successfully mapped detailed causal pathways spanning from sensory input to epigenetic regulation and microbiome dynamics, the pivotal finding is that the convergent gregarious phenotype across species masks fundamentally divergent genetic, neurochemical, and physiological mechanisms.

Direct comparisons between the two model species repeatedly reveal this divergence. In *L. migratoria*, gregarious nymphs produce PAN as an anti-cannibalistic pheromone (Chang et al., 2023a). In *S. gregaria*, however, PAN is exclusively released by gregarious mature males as a courtship-inhibiting pheromone and male-male repellent, a mechanism that enhances mate guarding to prevent sperm competition (Seidelmann & Ferenz, 2002; Seidelmann, Warnstorff & Ferenz, 2005). This functional divergence is further underscored by the essential role of the sensory protein SNMP1 in *S. gregaria*, where its loss impairs PAN detection and mating avoidance (Lehmann et al., 2024).

Neurochemical pathways show similar species-specificity. Serotonin drives rapid behavioral gregarization in *S. gregaria* (Anstey et al., 2009), yet enhances solitariness in *L. migratoria* (Guo, Ma & Kang, 2013). Adding to the complexity, one study in *S. gregaria* found no evidence for serotonin’s involvement in attraction-avoidance behavior (Tanaka & Nishide, 2013). Similarly, while octopamine has been implicated in phase-related behavior (Ma et al., 2015), an early study found no significant phase differences in octopamine levels in *S. gregaria*, directly contradicting prior reports (Morton & Evans, 1983).

The temporal dynamics of phase transition diverge as well. For example, *S. gregaria* exhibits rapid gregarization but slow solitarization (Roessingh & Simpson, 1994), while *L. migratoria* shows the opposite pattern (Guo et al., 2011), and *C. terminifera* changes rapidly in both directions (Gray et al., 2009). Remarkably, the Central American locust, *Schistocerca piceifrons* follows the *L. migratoria* pattern rather than that of its congener *S. gregaria* (Foquet et al., 2022), suggesting that phase-change kinetics are shaped by species-specific ecology and life-history rather than phylogenetic constraint alone.

Maternal effects on phase characteristics are well-established (Islam et al., 1994a, 1994b; Bouaïchi, Roessingh & Simpson, 1995; Mccaffery et al., 1998; Chapuis et al., 2008, 2010; Maeno & Tanaka, 2008; Wang et al., 2012; Chen et al., 2015b), with early work demonstrating that crowding during mating and oviposition alone is sufficient to produce gregarious offspring (Islam et al., 1994a, 1994b), an effect confirmed in natural populations and shown to accumulate across generations (Bouaïchi & Simpson, 2003). However, conflicting evidence persists regarding the timing of maternal programming (Maeno & Tanaka, 2010a cf. Nishide & Tanaka, 2019), the sensory requirements for transmission (Maeno, Tanaka & Harano, 2011 cf. Nishide & Tanaka, 2019), and the relative roles of egg chemistry (Mccaffery et al., 1998; Miller et al., 2008), yolk content (Maeno & Tanaka, 2009a), and epigenetic inheritance (Chen et al., 2015b; He et al., 2016; Robinson et al., 2016). This suggests that while the phenomenon is clear, its mechanistic basis is not, highlighting a key area for future research. Most comprehensively, transcriptomic analysis demonstrates that gene expression clusters more strongly by species than by phase (Bakkali et al., 2024). The majority of transcriptional responses to crowding are species-specific, with only a small set of “PhaseCore” genes showing conserved phase-related expression across both species (Yang et al., 2019a). This architecture—conserved gregarious phenotypes achieved through divergent molecular means—explains why behavioral and morphological similarities across locust species masks profound mechanistic differences.

However, this detailed mechanistic understanding comes with a critical caveat: the scarcity of field validation. Laboratory studies have successfully established causality and mapped mechanisms with precision unattainable in the field. Yet the ecological contexts that modulate phase change in nature, including variable climate, patchy resource distribution, predator pressure, and multi-species interactions, are deliberately stripped away in controlled designs. The rare studies that have integrated laboratory and field approaches demonstrate that these contexts matter profoundly (Lavy et al., 2019; Cease et al., 2023). This creates what we term a translational impasse: profound mechanistic knowledge exists parallel to, yet largely disconnected from, the ecological and evolutionary frameworks required for predictive application. This impasse is not merely an academic concern. Locust outbreaks continue to threaten food security across multiple continents, and effective management requires predictive tools that can anticipate gregarization before swarm form, as well as intervention strategies that are ecologically valid and species-appropriate. The contradictory findings reviewed here are not limitations of the evidence but rather critical indicators of the biological complexity that any successful translation must accommodate. They reinforce that interventions targeting phase change must be developed and validated with careful attention to species-specificity and ecological context, rather than assumed to operate universally.

### Management implications: bridging the translational impasse

The current preventive management paradigm relies on ecological forecasting that integrates remote sensing with field surveys to monitor vegetation greenness, rainfall patterns, and locust populations. This approach has proven valuable but operates on the principle that gregarization will occur when populations exceed carrying capacity, a threshold that, as the evidence shows, is itself modulated by vegetation structure, nutritional landscape, and species-specific factors. The mechanistic insights reviewed here offer opportunities to enhance this paradigm, but translation requires strategic integration rather than replacement. One pathway forward involves the development of physiological biomarkers that could provide earlier warning of incipient gregarization than density estimates alone. Gene expression signatures, neurochemical profiles, or microbiome characteristics associated with the early stages of phase transition, if they can be reliably detected in field-sampled individuals, might signal increasing gregarization risk before traditional indicators trigger intervention. The field-validated density thresholds and predictive models already in use (Cisse et al., 2013, 2015a; Cissé et al., 2016) provide a foundation for integrating such biomarkers into existing monitoring frameworks. Quantitative regression models developed for phase categorization (Martín-Blázquez & Bakkali, 2017; Saadi et al., 2024) offer standardized tools that could potentially bridge laboratory and field assessments.

For direct intervention, the evidence points away from searching for a universal silver bullet that would disrupt phase change across all locust species. The species-specificity of pheromone systems, neurochemical cascades, and even coloration mechanisms means that a tactic validated in one species cannot be assumed effective in another. Instead, translation requires a comparative functional framework that identifies vulnerable nodes within the phase-change networks of high-priority target species, then develops interventions tailored to those specific systems. The successful development of semiochemical-based approaches, including the use of PAN to solitarize gregarious hopper bands in the field (Bashir & Hassanali, 2010), demonstrates the feasibility of this approach when grounded in ecological understanding of the target species. Biological control agents offer another promising avenue, particularly those with dual-action effects. Entomopathogenic fungi such as *Metarhizium anisopliae* and microsporidia such as *Paranosema locustae* not only cause mortality but actively shift gregarious locusts toward solitary behavior (Shi et al., 2014; Feng et al., 2015; Li et al., 2020b). Understanding the mechanisms underlying these behavioral effects, whether through direct physiological impacts, modulation of the gut microbiome, or other pathways, could enable optimization of these agents for species-specific application. Crucially, all such translational efforts must be embedded within the existing preventive paradigm rather than positioned as replacements. The goal is enhancement: earlier warning through physiological biomarkers, more targeted intervention through species-specific disruptors, and validation of novel approaches through rigorous field testing that accounts for the ecological complexity that laboratory studies necessarily simplify.

### Limitations of the evidence

The conclusions of this review must be interpreted in light of several limitations inherent to the evidence base. First, the literature exhibits a severe taxonomic concentration, with 93.8% of studies focused on at least one of the two model species—*S. gregaria* and *L. migratoria*—and only 25 studies (6.2%) examining other species exclusively. Consequently, the current mechanistic understanding of locust phase polyphenism is, to a very large degree, an understanding of these two species. The substantial mechanistic divergence documented even between *S. gregaria* and *L. migratoria* (Bakkali et al., 2024) strongly suggests that findings from these models cannot be safely extrapolated to other locusts or grasshoppers with density-dependent plasticity, yet the limited evidence base on non-model species precludes confident generalization in either direction.

Second, the evidence is heavily skewed by study design, with laboratory experiments accounting for 339 studies (84.8% of the total) while field studies are far rarer, comprising only 24 studies (6%). The dominance of laboratory work, while essential for establishing causality under controlled conditions, inherently limits the external validity of the findings; the precise pathways and threshold effects mapped in simplified laboratory settings may operate differently under the complex, variable conditions of natural environments. The critical step of explicitly testing laboratory-derived mechanisms in the field remains a significant gap, as such validation studies are exceptionally rare. Third, while the field has shown a clear trajectory of increasing methodological rigor over time, the evidence base spans a century of research, and we cannot rule out the possibility of publication bias toward positive results. However, the past three decades are characterized by a high preponderance of “Low” risk of bias studies, lending confidence to the more recent findings.

### Limitations of the review processes

While this review adhered to PRISMA guidelines to ensure a systematic search and selection of literature, several methodological constraints of this review itself must be acknowledged. These limitations also highlight specific opportunities for strengthening future evidence syntheses in this field. The high heterogeneity in species, interventions, and measured outcomes across the 400 included studies precluded formal meta-analysis. While the narrative synthesis approach allows integration of complex, multifaceted evidence, it limits the statistical rigor and quantitative conclusiveness of our findings. Our search strategy, confined to major English-language databases, likely underrepresented non-English and grey literature. Incorporating regional databases and multilingual searches would enrich future syntheses. Additionally, despite careful design, our search strings may not capture all relevant studies due to terminological evolution across disciplines and decades.

### A strategic framework for future research

The patterns identified in this review directly inform a strategic framework for future research. To bridge the translational divide, the field must move beyond its current silos through four interconnected priorities, each designed to address a specific gap in the evidence base. First, expand the taxonomic and phylogenetic scope of mechanistic research. The finding that even two congeneric species, *S. gregaria* and *S. piceifrons*, exhibit different phase-change kinetics (Foquet, Castellanos & Song, 2021), and that transcriptomes cluster by species rather than phase (Bakkali et al., 2024), demands a decisive move beyond the current model-centric focus. A phylogenetically broad approach, including genome sequencing and functional genomics of non-model, high-impact pest species is essential to distinguish evolutionarily conserved core mechanisms from lineage-specific adaptations. Reporting of negative results and species-specific null findings must be actively encouraged to correct the current publication bias.

Second, deliberately integrate mechanistic and ecological research through field-laboratory hybrids. This requires manipulative field experiments that test laboratory-identified mechanisms under natural conditions, measuring how variables such as vegetation structure, climate variability, and community context modulate pathway function. It also requires laboratory experiments that incorporate ecological complexity, for example by using field-collected individuals, varying resource distribution, or simulating natural temperature regimes. The predictive model, which integrated density, vegetation, and ground cover to achieve high phase-prediction accuracy (Cissé et al., 2016), exemplifies the power of such integration. Future research should aim to embed physiological and molecular measurements within similar ecologically grounded frameworks.

Third, validate emerging regulatory mechanisms for their biocontrol potential in ecologically relevant contexts. The gut microbiome and epigenetic regulation represent promising but still nascent areas of investigation. The findings that gut bacteria produce aggregation pheromone components (Dillon, Vennard & Charnley, 2002) and that microbiome composition differs between phases (Lavy et al., 2019, 2022) suggest potential intervention points, but causal experiments establishing whether microbiome manipulation can prevent or reverse gregarization are lacking. Similarly, the demonstration that DNA methyltransferase 3 (Dnmt3) knockdown reduces gregarious locomotion (Hou et al., 2017) and that a nuclear-enriched long noncoding RNA (lncRNA), phenylalanine hydroxylase (PAHAL) can regulate dopamine synthesis (Zhang et al., 2020) points to epigenetic targets, but whether these mechanisms operate in non-model species or under field conditions remains unknown. Validation requires causal experiments in ecologically relevant settings, testing across multiple species to assess conservation, and explicit consideration of whether identified targets are druggable or otherwise amenable to intervention in natural populations.

Fourth, embed translation within the preventive paradigm through species-specificity and equitable partnership. Translation should be conceptualized not as a separate phase of research but as an integral component of the research agenda from the outset. This means designing basic research with eventual application in mind: prioritizing field validation for the two model species while simultaneously building foundational mechanistic knowledge for understudied pest species, developing field-applicable physiological biomarkers, testing semiochemical interventions in complex field settings, and evaluating biological control agents for their effects on phase status and swarm formation. Critically, this translational agenda requires equitable transdisciplinary partnerships with researchers and practitioners in outbreak-affected regions. Local knowledge of outbreak dynamics, ecological context, and management feasibility is essential for designing interventions that are not only scientifically sound but practically implementable. Building research capacity in locust-affected countries ensures that those most in need benefit from mechanistic insight.

## Conclusion

This systematic review synthesizes over a century of research on locust phase polyphenism, revealing a field at a critical juncture. A deep but narrow understanding of proximate mechanisms remains largely disconnected from the ecological and evolutionary frameworks required for predictive application. The central insight emerging from this synthesis is that the remarkable phenotypic convergence of swarming locusts, the shared outcome of gregarious behavior and morphology across species, is not the product of a single universal molecular script. Rather, it emerges from diverse genetic, neurochemical, and physiological orchestrations that have evolved within specific lineages. This finding fundamentally reframes how we should interpret the mechanistic literature and how we should approach translation.

The translational impasse identified here, entrenched by taxonomic narrowness, methodological isolation, and fragmentation across levels of analysis, constitutes the central challenge. To bridge this divide, future progress must be governed by a new, integrative paradigm. This paradigm explicitly recognizes that mechanisms are lineage-specific adaptations and that laboratory findings, however causally rigorous, require ecological validation before they can inform management. The path forward lies not in further deepening the silo of a single model system, but in systematically harnessing comparative functional biology across a representative phylogenetic spectrum. Applying the resolving power of modern genomics, causal genetics, and microbiome manipulation to both established and understudied locust species will rigorously disentangle evolutionarily conserved core mechanisms from lineage-specific innovations.

Crucially, this mechanistic knowledge must then be deliberately integrated with field-based climate ecology and socio-economic realities through equitable, transdisciplinary partnerships. This integration is the essential catalyst that transforms fundamental insight into actionable management tools, enabling the development of predictive physiological biomarkers for early warning, the rational design of species-specific behavioral disruptors, and the validation of next-generation biocontrol agents that target the plasticity enabling swarm formation itself. By adopting this strategic, comparative, and partnership-based agenda, the profound biological knowledge of locust phase change can be decisively leveraged to generate the predictive, sustainable, and context-aware strategies necessary to safeguard global food security in an era of rapid environmental change.

## Supplemental Information

10.7717/peerj.21374/supp-1 Supplemental Information 1 PRISMA Checklist.

10.7717/peerj.21374/supp-2 Supplemental Information 2 Search strings.

10.7717/peerj.21374/supp-3 Supplemental Information 3 Complete lists of included studies.

10.7717/peerj.21374/supp-4 Supplemental Information 4 Rationale and contributions.

## Additional Information and Declarations

### Data Availability

The following information was supplied regarding data availability:

This is a Systematic Review/Meta-analysis.
