# Supplementary material for: A systematic review of locust phase polyphenism: from proximate mechanisms to ecology and management
Source: PeerJ. 2026 Jul 7;14:e21374. doi: 10.7717/peerj.21374 (PMC13353236; doi:10.7717/peerj.21374)
Supplement: Supplemental Information 1 [file peerj-14-21374-s001.docx]

| **Section and Topic** | **Item #** | **Checklist item** | Location where item is reported |
| --- | --- | --- | --- |
| **TITLE** | | |  |
| Title | 1 | Identify the report as a systematic review. | Title: A Systematic Review of Locust Phase Polyphenism: From Proximate Mechanisms to Ecology and Management |
| **ABSTRACT** | | |  |
| Abstract | 2 | See the PRISMA 2020 for Abstracts checklist. | Abstract (The abstract includes all key elements: objectives, methods, results, discussion.) |
| **INTRODUCTION** | | |  |
| Rationale | 3 | Describe the rationale for the review in the context of existing knowledge. | Introduction (Paragraphs 1-2) |
| Objectives | 4 | Provide an explicit statement of the objective(s) or question(s) the review addresses. | Introduction (Final paragraph) |
| **METHODS** | | |  |
| Eligibility criteria | 5 | Specify the inclusion and exclusion criteria for the review and how studies were grouped for the syntheses. | 2.3. Study Selection, Data Extraction and Risk of Bias Assessment |
| Information sources | 6 | Specify all databases, registers, websites, organisations, reference lists and other sources searched or consulted to identify studies. Specify the date when each source was last searched or consulted. | 2.1. Literature Search Strategies (Databases named; search date: "1921 to February 2025") |
| Search strategy | 7 | Present the full search strategies for all databases, registers and websites, including any filters and limits used. | 2.1. Literature Search Strategies (Provides key search terms, Boolean operators, and filters used.). Supplementary File 1 |
| Selection process | 8 | Specify the methods used to decide whether a study met the inclusion criteria of the review, including how many reviewers screened each record and each report retrieved, whether they worked independently, and if applicable, details of automation tools used in the process. | 2.2. Study Selection and Screening Process (Two independent reviewers, process for resolving disagreements, use of R packages for deduplication.) |
| Data collection process | 9 | Specify the methods used to collect data from reports, including how many reviewers collected data from each report, whether they worked independently, any processes for obtaining or confirming data from study investigators, and if applicable, details of automation tools used in the process. | 2.3. Study Selection, Data Extraction and Risk of Bias Assessment |
| Data items | 10a | List and define all outcomes for which data were sought. Specify whether all results that were compatible with each outcome domain in each study were sought (e.g. for all measures, time points, analyses), and if not, the methods used to decide which results to collect. | 2.3. Study Selection, Data Extraction and Risk of Bias Assessment |
|  | 10b | List and define all other variables for which data were sought (e.g. participant and intervention characteristics, funding sources). Describe any assumptions made about any missing or unclear information. | 2.3. Study Selection, Data Extraction and Risk of Bias Assessment |
| Study risk of bias assessment | 11 | Specify the methods used to assess risk of bias in the included studies, including details of the tool(s) used, how many reviewers assessed each study and whether they worked independently, and if applicable, details of automation tools used in the process. | 2.3. Study Selection, Data Extraction and Risk of Bias Assessment |
| Effect measures | 12 | Specify for each outcome the effect measure(s) (e.g. risk ratio, mean difference) used in the synthesis or presentation of results. | 2.4. Data Synthesis methods (A meta-analysis was not performed; a narrative synthesis was conducted. Therefore, effect measures are not applicable.) |
| Synthesis methods | 13a | Describe the processes used to decide which studies were eligible for each synthesis (e.g. tabulating the study intervention characteristics and comparing against the planned groups for each synthesis (item #5)). | 2.3. Study Selection, Data Extraction and Risk of Bias Assessment |
|  | 13b | Describe any methods required to prepare the data for presentation or synthesis, such as handling of missing summary statistics, or data conversions. | 2.4. Data Synthesis methods (Describes standardization of metadata and data processing in R.) |
|  | 13c | Describe any methods used to tabulate or visually display results of individual studies and syntheses. | 2.4. Data Synthesis methods |
|  | 13d | Describe any methods used to synthesize results and provide a rationale for the choice(s). If meta-analysis was performed, describe the model(s), method(s) to identify the presence and extent of statistical heterogeneity, and software package(s) used. | 2.4. Data Synthesis methods (Explicitly states narrative synthesis was used due to high heterogeneity; rationale provided.) |
|  | 13e | Describe any methods used to explore possible causes of heterogeneity among study results (e.g. subgroup analysis, meta-regression). | Not Applicable. A meta-analysis was not performed, so statistical heterogeneity was not assessed. Thematic analysis serves this purpose qualitatively |
|  | 13f | Describe any sensitivity analyses conducted to assess robustness of the synthesized results. | Not Applicable. |
| Reporting bias assessment | 14 | Describe any methods used to assess risk of bias due to missing results in a synthesis (arising from reporting biases). | 2.3. Study Selection, Data Extraction and Risk of Bias Assessment |
| Certainty assessment | 15 | Describe any methods used to assess certainty (or confidence) in the body of evidence for an outcome. | Not Applicable. |
| **RESULTS** | | |  |
| Study selection | 16a | Describe the results of the search and selection process, from the number of records identified in the search to the number of studies included in the review, ideally using a flow diagram. | 3.1. Literature search and screening outcomes (and reference to Figure 1) |
|  | 16b | Cite studies that might appear to meet the inclusion criteria, but which were excluded, and explain why they were excluded. | 3.1. Literature search and screening outcomes; Figure1 |
| Study characteristics | 17 | Cite each included study and present its characteristics. | 3.2 Characteristics of Included Studies (see also supplementary file 2) |
| Risk of bias in studies | 18 | Present assessments of risk of bias for each included study. | 3.3. Risk of Bias within Studies (Provides a qualitative summary of the risk of bias) |
| Results of individual studies | 19 | For all outcomes, present, for each study: (a) summary statistics for each group (where appropriate) and (b) an effect estimates and its precision (e.g. confidence/credible interval), ideally using structured tables or plots. | Not Applicable in a standard form. Key findings from individual studies are integrated into the narrative synthesis in Section 3.4 |
| Results of syntheses | 20a | For each synthesis, briefly summarise the characteristics and risk of bias among contributing studies. | 3.4. Thematic Synthesis. |
|  | 20b | Present results of all statistical syntheses conducted. If meta-analysis was done, present for each the summary estimate and its precision (e.g. confidence/credible interval) and measures of statistical heterogeneity. If comparing groups, describe the direction of the effect. | statistical syntheses conducted (Section 3.2); No meta-analysis was performed; results are presented narratively. |
|  | 20c | Present results of all investigations of possible causes of heterogeneity among study results. | Not Applicable. |
|  | 20d | Present results of all sensitivity analyses conducted to assess the robustness of the synthesized results. | Not Applicable. No sensitivity analyses were performed. |
| Reporting biases | 21 | Present assessments of risk of bias due to missing results (arising from reporting biases) for each synthesis assessed. | 3.3. Risk of Bias within Studies |
| Certainty of evidence | 22 | Present assessments of certainty (or confidence) in the body of evidence for each outcome assessed. | Not Applicable. (See item #15) |
| **DISCUSSION** | | |  |
| Discussion | 23a | Provide a general interpretation of the results in the context of other evidence. | 4.1. General Interpretation of the Results |
|  | 23b | Discuss any limitations of the evidence included in the review. | 4.3. Limitations of the Evidence |
|  | 23c | Discuss any limitations of the review processes used. | 4.4. Limitations of the Review Processes |
|  | 23d | Discuss implications of the results for practice, policy, and future research. | 4.5 A Strategic Framework for Future Research |
| **OTHER INFORMATION** | | |  |
| Registration and protocol | 24a | Provide registration information for the review, including register name and registration number, or state that the review was not registered. | Not registered |
|  | 24b | Indicate where the review protocol can be accessed, or state that a protocol was not prepared. | Not applicable. |
|  | 24c | Describe and explain any amendments to information provided at registration or in the protocol. | Not applicable. |
| Support | 25 | Describe sources of financial or non-financial support for the review, and the role of the funders or sponsors in the review. | Competing Interests/Funding section: "This research received no external funding. |
| Competing interests | 26 | Declare any competing interests of review authors. | Competing Interests section |
| Availability of data, code and other materials | 27 | Report which of the following are publicly available and where they can be found: template data collection forms; data extracted from included studies; data used for all analyses; analytic code; any other materials used in the review. | Data is uploaded as a Supplemental file 2 |

*From:*  Page MJ, McKenzie JE, Bossuyt PM, Boutron I, Hoffmann TC, Mulrow CD, et al. The PRISMA 2020 statement: an updated guideline for reporting systematic reviews. BMJ 2021;372:n71. doi: 10.1136/bmj.n71. This work is licensed under CC BY 4.0. To view a copy of this license, visit <https://creativecommons.org/licenses/by/4.0/>
