# Supplementary material for: A systematic review of locust phase polyphenism: from proximate mechanisms to ecology and management
Source: PeerJ. 2026 Jul 7;14:e21374. doi: 10.7717/peerj.21374 (PMC13353236; doi:10.7717/peerj.21374)
Supplement: Supplemental Information 2 [file peerj-14-21374-s002.docx]

**Lists of search strings used across each database**

**Web of Sciences**

("locust" OR "Schistocerca" OR "Locusta migratoria" OR "Nomadacris" OR "Chortoicetes"OR "Oedaleus" OR "Austracris") AND ("phase polyphenism" OR "phase polymorphism" OR "phase change" OR "phase transition" OR "phenotypic plasticity" OR "density-dependent" OR "gregari*" OR "solitar*" OR "phase characteristic*") AND ("behavi*" OR "morphometr*" OR "colo*" OR "pheromone" OR "juvenile hormone" OR "corazonin" OR "serotonin" OR "transcriptom*" OR "microbiome").

**Scopus**

TITLE-ABS-KEY ( "Locust" OR " Schistocerca" OR "Locusta migratoria" OR "Nomadacris" OR "Chortoicetes" OR "Oedaleus" OR “Austracris” ) AND TITLE-ABS-KEY ( "phase polyphenism" OR "Phenotypic plasticity" OR " phase polymorphism" OR "density-dependent" OR "phase change" OR "gregari*" OR "solitar*" ) AND TITLE-ABS-KEY ( "behavio*" OR "morphometr*" OR "juvenile hormone" OR "corazonin" OR "transcriptom*" OR "microbiome") AND ( LIMIT-TO ( SUBJAREA , "AGRI" ) OR LIMIT-TO ( SUBJAREA , "NEUR" ) OR LIMIT-TO ( SUBJAREA , "BIOC" ) OR LIMIT-TO ( SUBJAREA , "IMMU" ) ) AND ( LIMIT-TO ( DOCTYPE , "ar" ) ) AND ( LIMIT-TO ( LANGUAGE , "English" ) ).

**PubMed**

(Locust* OR Schistocerca OR "Locusta migratoria" OR Nomadacris OR Chortoicetes OR Oedaleus OR Austracris) AND (phase polyphenism OR "phenotypic plasticity" OR density-dependent OR gregarious OR solitary OR solitarisation) AND (behavior* OR morphometr* OR "juvenile hormone" OR corazonin OR transcriptom* OR microbiome).
