# Supplementary material for: A systematic review of locust phase polyphenism: from proximate mechanisms to ecology and management
Source: PeerJ. 2026 Jul 7;14:e21374. doi: 10.7717/peerj.21374 (PMC13353236; doi:10.7717/peerj.21374)
Supplement: Supplemental Information 4 [file peerj-14-21374-s004.docx]

**Rationale and Contribution of our Systematic Review**

**1. Rationale for the Systematic Review**

Locust phase polyphenism represents a quintessential example of phenotypic plasticity, with profound implications for both evolutionary biology and global food security. Over a century of research has progressively unveiled the mechanisms underlying the density-dependent shift from solitary, cryptic individuals to gregarious, swarming pests. However, this extensive effort has generated a rich but deeply fragmented body of literature, characterized by isolated advancements across molecular biology, ecology, neuroethology, and microbiology. While numerous authoritative reviews have synthesized specific facets of this phenomenon, they have remained predominantly qualitative, discipline-specific, and narrative in approach. Consequently, a critical synthesis is conspicuously absent: a systematic, quantitative integration of the entire research landscape is urgently required to diagnose the field's trajectory, expose its foundational biases, and chart a definitive, evidence-based path forward.

This systematic review addresses three urgent and interconnected needs:

First, to quantify systemic imbalances that constrain understanding. The existing knowledge base suffers from significant, unquantified biases that limit both biological inference and translational application. Our analysis reveals a pronounced taxonomic skew, with 93.8% of studies focused on at least one of just two model species—Schistocerca gregaria and Locusta migratoria—leaving the mechanistic diversity of non-model locusts critically underexplored. A stark methodological divide compounds this limitation: laboratory-based studies account for 84.8% of the literature, while research integrating ecological complexity and field-based validation constitutes a mere 12.2%. This dual fragmentation—taxonomic narrowness coupled with methodological isolation—fundamentally hinders holistic understanding and severely limits the translational potential of molecular discoveries for outbreak prediction and management.

**Second, to address an evolving threat landscape intensified by global change.** Climate change and land-use shifts are fundamentally altering locust population dynamics, geographical ranges, and outbreak frequency, creating unprecedented demand for predictive models that integrate environmental drivers with internal physiological mechanisms. While transformative technologies—such as CRISPR-Cas9 for functional genomics and remote sensing for landscape-scale ecology—offer new power to address these challenges, their adoption in locust research has been uneven and poorly coordinated across disciplines. A systematic assessment of current research trends is essential to evaluate whether the field is adequately positioned to harness these tools and to steer future inquiry toward a more technologically adept and genuinely interdisciplinary paradigm capable of meeting the escalating threat.

**Third, to critically evaluate emerging regulatory paradigms.** Novel mechanisms have recently emerged that challenge traditional neuro-endocrine frameworks of phase regulation. The role of the gut microbiome in driving aggregation behavior and the contribution of transgenerational epigenetics in priming phase change across generations demand rigorous evaluation of their causal significance. These paradigms remain largely correlational, and their functional relevance under ecologically realistic conditions is unknown. This review moves beyond descriptive synthesis to critically appraise whether these emerging mechanisms represent genuine regulatory nodes—and therefore potential targets for sustainable management—or merely epiphenomena of the crowded condition.

**2. Contribution to Knowledge**

This study provides the **first comprehensive systematic review of locust phase polyphenism**, making several seminal contributions that fundamentally advance the field beyond all previous narrative syntheses:

**A paradigm shifts from narrative synthesis to evidence-based integration.** Unlike prior qualitative reviews, which have synthesized knowledge within disciplinary silos, this study establishes a new empirical foundation by systematically aggregating and critically appraising 400 studies spanning 1921–2025. We apply rigorous, reproducible methods—including PRISMA-guided search strategies, dual-reviewer screening, formal risk-of-bias assessment, and structured narrative synthesis—to generate conclusions that are transparent, auditable, and quantitatively grounded. This approach transforms the evidence base from a collection of authoritative opinions into a systematically mapped intellectual landscape.

**Identification and diagnosis of systemic biases with quantitative precision.** We provide the first quantitative evidence of the field's core limitations, revealing patterns previously suspected but never empirically documented. We demonstrate the extreme dominance of model species (93.8% of studies), the marginalization of research from non-model outbreak species (6.2%), and the profound methodological divide between laboratory and field investigation (84.8% vs. 12.2%). Beyond these descriptive findings, we establish their consequences: direct species comparisons reveal fundamental mechanistic divergence—in neurochemical cascades, pheromone systems, transition kinetics, and transcriptomic architecture—demonstrating that the convergent gregarious phenotype masks deeply divergent molecular mechanisms. This diagnosis provides an irrefutable, evidence-based rationale for expanding phylogenetic breadth and integrating ecological complexity into future research.

**Integrated synthesis of emerging mechanisms within a unified framework.** This review is the first to systematically incorporate and critically appraise rapidly expanding findings on the gut microbiome and epigenetic regulation, positioning them not as isolated curiosities but as central, interactive components within the broader neuro-endocrine cascade governing phase transition. We synthesize evidence that gut bacteria produce aggregation pheromone precursors, that DNA methylation and non-coding RNAs functionally regulate gregarious behavior, and that these mechanisms may interface with transgenerational transmission of phase state. By embedding these emerging paradigms within the established mechanistic architecture, we provide a comprehensive map of known regulators and, crucially, identify where causal validation remains lacking.

**Identification of a translational impasse with profound management implications.** Our synthesis reveals that profound mechanistic knowledge exists in parallel to—yet remains largely disconnected from—the ecological and evolutionary frameworks required for predictive application. We term this the "translational impasse": laboratory-derived pathways, however causally rigorous, have rarely been tested under the variable, complex conditions of natural environments where outbreaks actually occur. This finding fundamentally reframes how the field should approach translation, moving the discourse from "what mechanisms exist" to "which mechanisms operate under ecologically relevant conditions and can be exploited for management."

**A strategic framework to bridge the translational divide.** In direct response to our diagnostic findings, we propose a forward-looking, actionable research agenda explicitly designed to overcome identified biases. This roadmap prioritizes: (1) phylogenetically broad comparative multi-omics to distinguish evolutionarily conserved cores from lineage-specific adaptations; (2) integrated field-laboratory experiments that deliberately incorporate climate variability, landscape heterogeneity, and nutritional ecology; (3) causal validation of emerging regulators (microbiome, epigenetics) through manipulative experiments under ecologically realistic conditions; and (4) equitable, transdisciplinary partnerships with researchers and practitioners in outbreak-affected regions to ensure that mechanistic insights translate into context-appropriate management tools. This framework transforms diagnosis into prescription, providing the field with a clear, evidence-based path toward predictive, sustainable management strategies in an era of rapid environmental change.
